# Identification of potential binding pocket on viral oncoprotein HPV16 E6: a promising anti-cancer target for small molecule drug discovery

**DOI:** 10.1186/s12860-019-0214-3

**Published:** 2019-08-06

**Authors:** Srikanth Kolluru, Rosemary Momoh, Lydia Lin, Jayapal Reddy Mallareddy, John L. Krstenansky

**Affiliations:** Keck Graduate Institute School of Pharmacy, 535 Watson Dr, Claremont, CA 91711 USA

**Keywords:** Human papilloma virus, HPV E6, Myricetin, Flavonol, Molecular modelling

## Abstract

**Background:**

Several human cancers, especially cervical cancer are caused by the infection of high risk strains of human papillomaviruses (HPV), notably HPV16. It is implicated that the oncoprotein E6 expressed from HPV, is inhibiting the apoptotic pathway by binding to adaptor molecule FADD (Fas-associated death domain). Inhibiting E6 interactions with FADD could provide a promising treatment for cervical cancer. There are few small molecules reported to inhibit such interactions. However, the FADD binding site information on the HPV E6 is not currently available. This binding site information may provide an opportunity to design new small molecule inhibitors to treat E6 mediated cancers. In this study we report the possible binding pocket on HPV16 E6 oncoprotein by using activity data of reported inhibitors through a stepwise molecular modeling approach.

**Results:**

Blind docking and removing duplicates followed by visual inspection to determine ligand-receptor interactions provided 68 possible binding sites on the E6 protein. Individual docking of all known inhibitors lead to the identification of 28 pockets having some kind of correlation with their activity data. It was also observed that several of these pockets overlapped with each other, having some amino acids in common. Amino acids Leu50 and Cys51 were identified as key E6 residues for high affinity ligand binding which are seen in most of these pockets. In most cases, ligands demonstrated a hydrogen bond interaction with Cys51. Ala61, Arg131 and Gln107 were also frequently observed showing interactions among these pockets. A few amino acids unique to each ligand were also identified representing additional interactions at the receptor site.

**Conclusions:**

After determining receptor-ligand interactions between E6 oncoprotein and the six known inhibitors, the amino acids Cys51, Leu50, Arg102, Arg131, Leu67, Val62, and Gln107 were identified to have importance in E6 inhibition. It was generally observed that Leu50 and Cys51 are necessary for high binding affinity with Cys51 being essential for hydrogen bonding. This study identified a potential binding pocket for the E6 inhibitors. Identification of the ligand binding pocket helps to design novel inhibitors of HPV16 E6 oncoprotein as a promising treatment for cervical cancer.

**Electronic supplementary material:**

The online version of this article (10.1186/s12860-019-0214-3) contains supplementary material, which is available to authorized users.

## Background

Human papillomavirus (HPV) infection is one of the major causes of cervical cancer in women worldwide [1–3]. To date, more than 200 different HPVs have been characterized, and new types are regularly being added. These viruses are sub grouped into mucosal and cutaneous HPVs according to their ability to infect the mucosa or the skin of genital or upper respiratory tracts. Within each of these HPV groups, they are further sub-classified based on their risk to cause malignancy [4]. Of all, HPV16 is known to be the most oncogenic type within the high-risk group [5, 6]. Prophylactic HPV vaccines are currently available, which help control or prevent certain types of cancers including cervical cancers. However, these vaccines are very expensive and have no utility in already infected patients [7, 8].

**Fig. 1:**
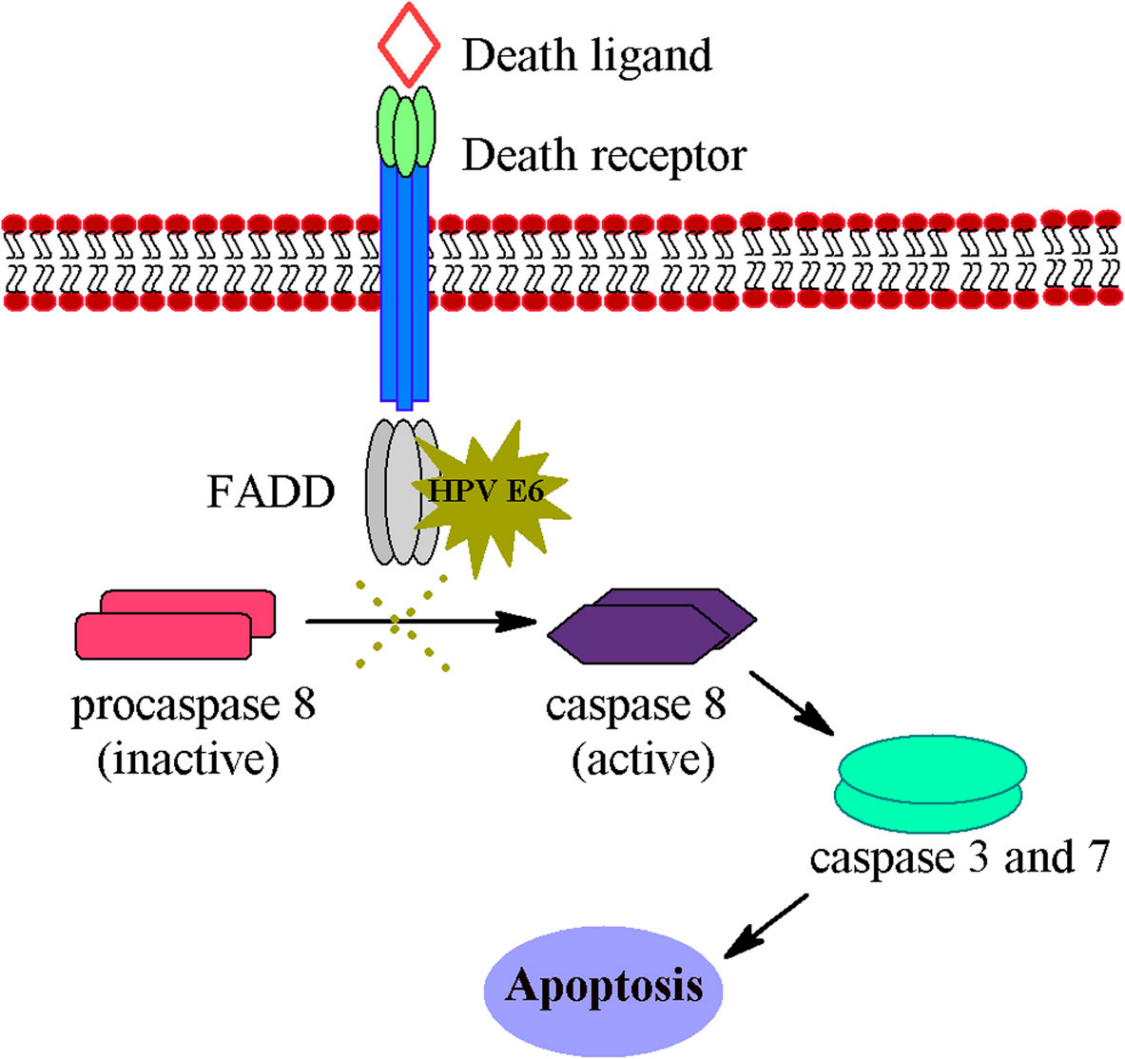
Schematic representation of extrinsic apoptotic pathway and HPV E6 blockade of FADD mediated apoptosis. Under normal conditions, binding of death ligands such as FasL, TNF, TRAIL to their receptors e.g., Fas, TNF-R, TRAIL-R initiates an extrinsic apoptotic pathway. This leads to the association of adaptor molecules such as FADD, TRADD to the death receptor forming a death inducing signaling complex (DISC), which activates procaspase 8. Activated procaspase 8 releases active cysteine-aspartic proteases that enable caspase 8 to cleave and activate effector caspases 3 and 7. However, in the case of HPV E6 oncoprotein expressed cells this sequence cannot occur. HPV E6 binds to FADD blocking procaspase 8 activation and subsequent actions. This binding also leads to the degradation of FADD. Both of these events prevent apoptosis and cells become resistant to cell death induced by TNF and TRAIL

The oncogenicity of HPV is associated with its high risk oncoproteins, E6 and E7. E6 interacts with a multitude of host cellular proteins such as p53, E6AP, MAML1, retinoblastoma family proteins and proteins containing PDZ domains. E6 inactivates these proteins and affects multiple cellular pathways such as cell proliferation and apoptosis [9]. One such significant interaction of HPV E6 oncoprotein induces tumor by interfering with the function of p53, a critical tumor suppressor protein. It induces proteasome-dependent p53 degradation, while E7 induces tumors by inactivating pRB protein [10]. In addition to p53, E6 also interacts with array of other cellular proteins [9, 11–13]. The E6 protein has the ability to prevent apoptosis of infected cells through its interaction with FADD and procaspase 8 in extrinsic apoptotic pathway (Fig. 1) [14–16]. The HPV infected cells will be resensitized to apoptosis by preventing the binding of E6 to FADD and procaspase 8 [14]. HPV E6-FADD/procaspase 8 interaction inhibitors could offer a promising therapy to treat HPV-associated cancers [17, 18].

A few small molecules inhibitors were developed via high throughput screening for HPV E6 and E6AP interaction, with only modest activities [7, 19]. A library of flavonols were reported as inhibitors of HPV16 E6 and FADD and procaspase 8 [20]. However, the specific binding site on the E6 protein is not known. Having the binding site information could provide insight for the rational design of improved inhibitors suitable for the treatment of HPV-mediated cancers. The development of small molecule inhibitors will provide a major advancement in the treatment of cervical cancers due to HPV. Over 99% of the patients having cervical lesions are positively identified with HPV DNA [2]. These could be better alternatives or treatment options for women in countries that do not have access to or cannot afford vaccines. In this study, we identified the ligand binding pocket on the HPV16 E6 oncoprotein by using activity data of reported inhibitors through a systematic molecular modeling study. HPV16 E6 inhibitors could make a promising prophylactic treatment to prevent cancers in HPV infected patients.

## Results

**Fig. 2:**
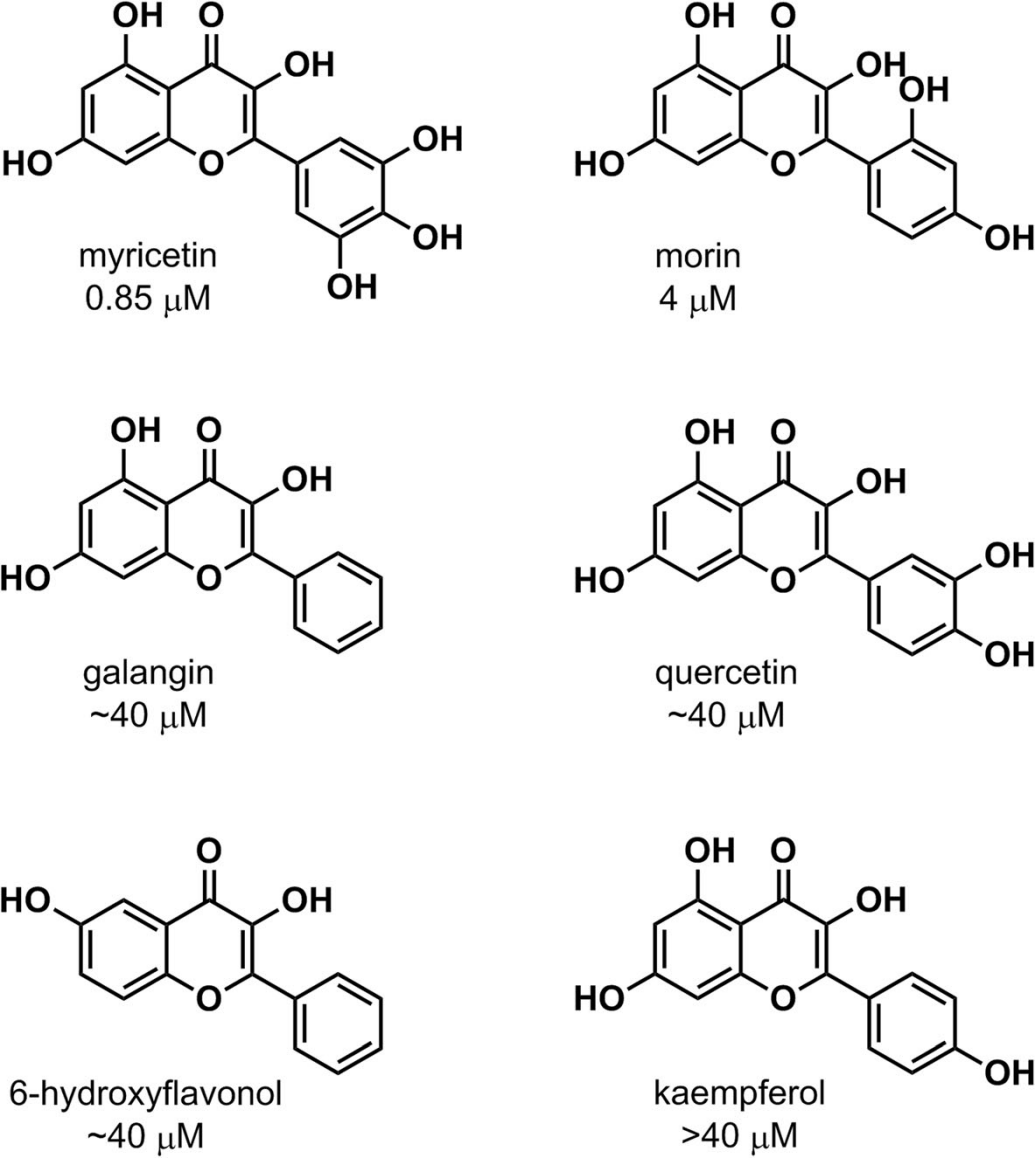
Structures and IC_50_s of 3-flavonol inhibitors of HPV16 E6 [20]

**Fig. 3:**
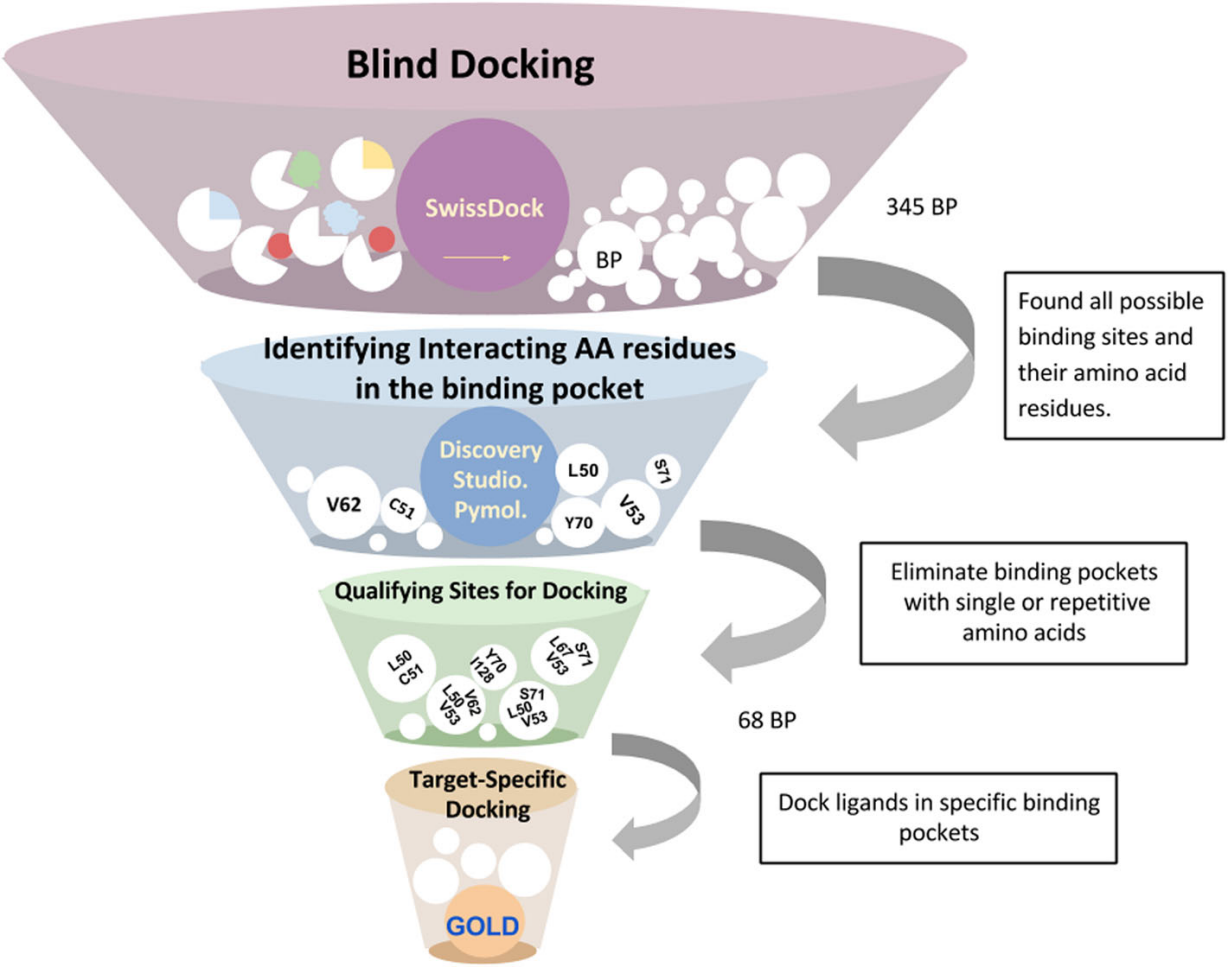
Flowchart showing overall methodology to identify potential ligand binding pocket(s) (BP) on the HPV16 E6 oncoprotein

The purpose of this study was to identify the flavonol binding pocket on the HPV16 E6 oncoprotein. The six flavonol inhibitors (Fig. 2) used in this study were reported by Yuan, et al. as having IC_50_ values ranging from 0.85 μM to > 40 μM [20]. It should be noted that the library screened (ActiTarg-K Library; TimTec LLC, Newark, DE [21]) contained flavones and other flavonols that did not demonstrate inhibition at these levels. The goal was to identify potential binding site based not only on the predicted affinity of the ligands, but also on their correlation with experimental relative affinities (IC_50_s). Knowledge of the binding site and nature of the interaction would provide valuable information for the rational design of potent and selective inhibitors of HPV16 E6. Figure 3 illustrates the overall strategy that was adopted to identify the binding pocket on HPV16 E6.

### Step 1: blind docking

In order to identify all pockets on the HPV16 E6 protein, blind docking was performed using a web-based program called SwissDock. The ligands myricetin, morin, galangin, kaempferol, 6-hydroxy flavonol, and quercetin (Fig. 2) were docked on chain ‘C’ from the unit cell of the crystal structure (PDB: 4GIZ). SwissDock generates all possible binding modes for each ligand and the most favorable binding modes at a given pocket were clustered. All ligand clusters were saved in an output file called “predictions file”. The predictions file provided Cluster Rank/Element Full Fitness and estimated binding free energy ΔG. Sample SwissDock predictions file data for the ligand morin docked in HPV16 E6, is shown in the Additional file 1: Figure S2, as viewed in Jmol applet within the SwissDock program. A cluster is a predicted binding pocket on the target protein, and the cluster rank/element represents the different conformations of the ligand in a certain cluster. Individual clusters and clusters with noticeably different conformations when viewed in SwissDock were saved separately from output predictions file for visualization. Overall, we identified 345 individual clusters after blind docking all six ligands.

### Step 2: cluster visualization to determining receptor-ligand interactions

Discovery Studio visualizer was used to visualize the receptor ligand interactions for all individual clusters obtained from Step 1. Each cluster for every ligand was inspected for amino acids interacting with the ligand, types of bonds formed, the specific atoms involved and the distance between them. All of the interacting amino acids with the target receptor were noted for each cluster.

**Fig. 4:**
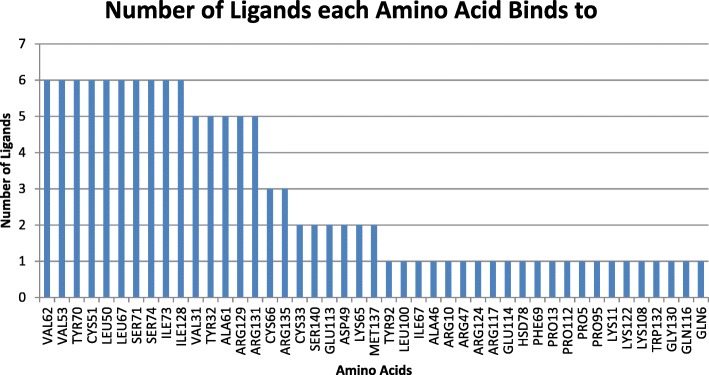
Amino acids identified from various clusters and number of ligands where they demonstrated interaction with the ligand

The most common ligand-interacting amino acids are shown in Fig. 4. There were 10 amino acids that showed interactions with all 6 ligands in either cluster. In other words, there was no instance where each ligand interacted with all 10 amino acids in one cluster. However, among the different clusters, Cys51 and Leu50 were seen very frequently. The location of these amino acids indicate that they are arranged in a large cavity on the E6 oncoprotein, which indicates potential site for a binding pocket.

### Step 3: removing duplicate clusters based on interacting amino acids

**Fig. 5:**
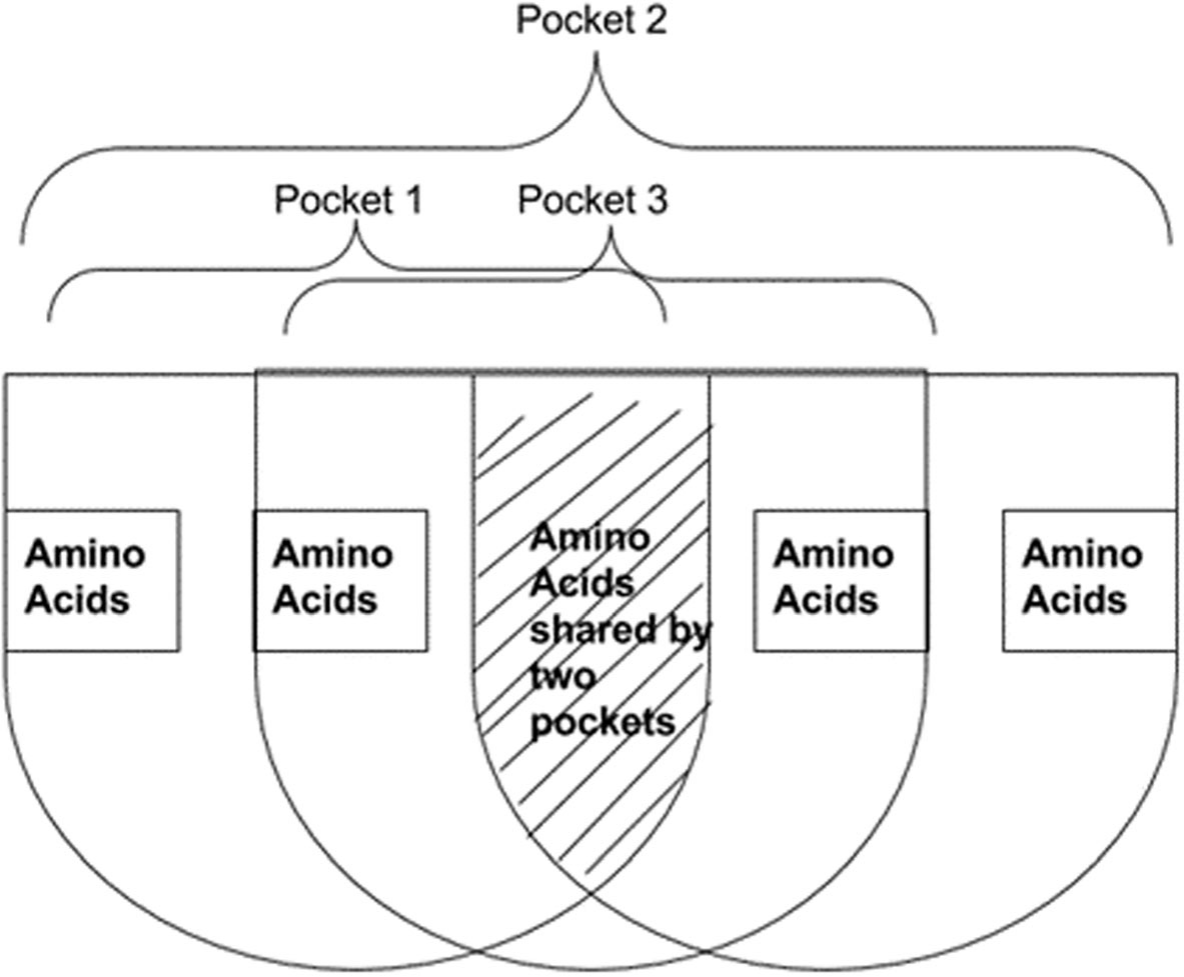
Multiple ligand clusters sharing common amino acids at the binding site indicating their close proximity

All six flavonols are expected to bind to the same pocket on the target protein. By analyzing the amino acids identified for each cluster for each ligand, all duplicates were eliminated. It was observed that some clusters had only one interacting amino acid; these were eliminated from the dataset. After careful observation, it was determined that several common amino acids are part of different clusters, indicating the close proximity of these individual binding pockets on the large binding cavity. Figure 5 depicts the close proximity of multiple binding pockets representing different clusters with common amino acids.

### Step 4: docking into individual binding pockets

**Fig. 6:**
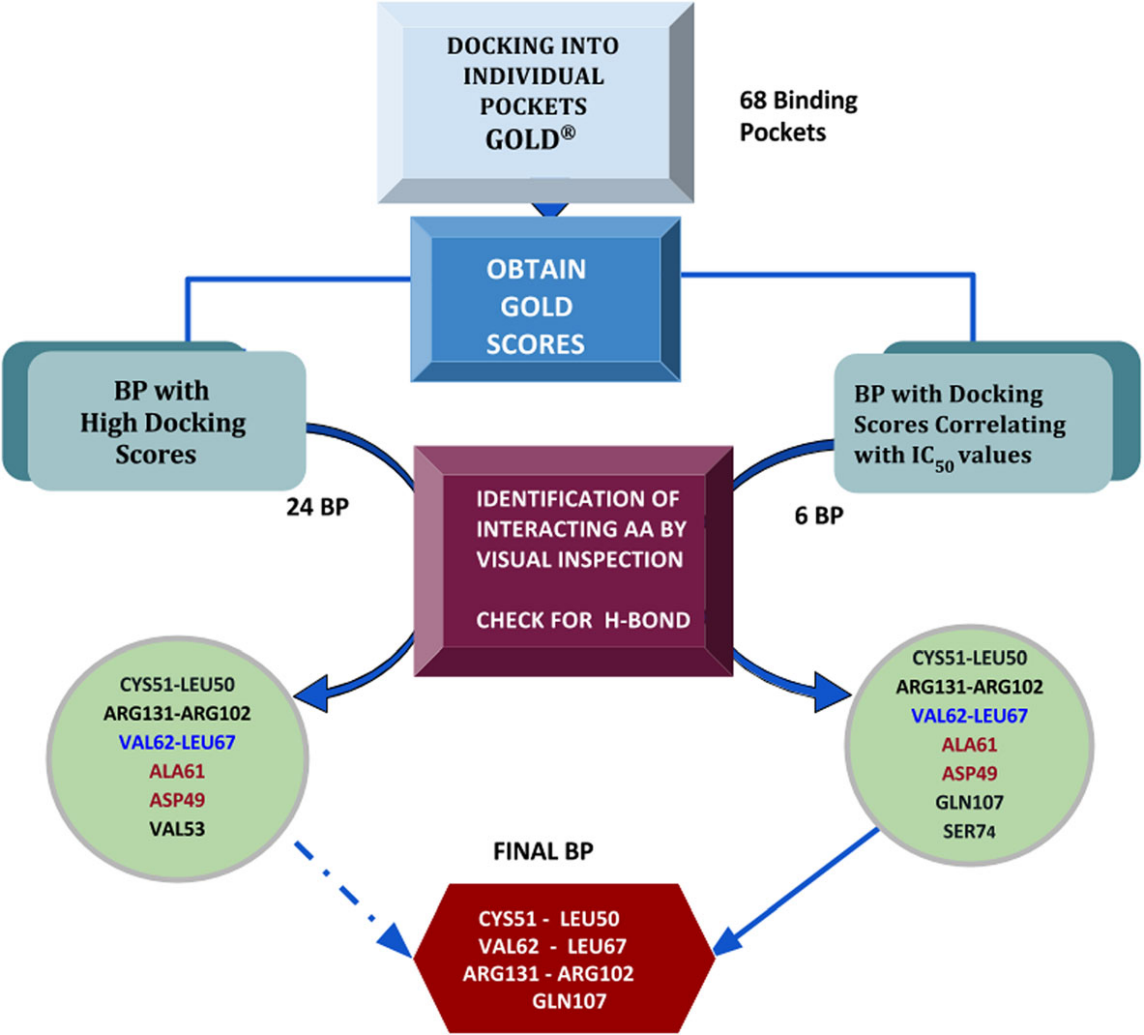
Schematic representation of steps from GOLD docking to predict potential binding pocket amino acid residues

By following the protocol in Step 3, 68 binding pockets were identified. It was observed that several of these clusters share multiple amino acids as illustrated in Fig. 6. All six ligands were docked individually into each of these 68 pockets on E6 oncoprotein using GOLD docking program to determine their relative affinities and to identify additional amino acids that may have a role in ligand binding. Binding sites were defined using two or more amino acids residues obtained from the previous step. Twenty four of the 68 potential binding pockets had docking scores greater than 40. GOLD docking scores for all ligands are shown in Additional file 1: Table S1. Twenty four pockets showed high docking scores of 40 or higher, representing higher affinity to ligands. Six pockets showed docking scores correlating well with the inhibitory activities. Receptor-ligand interactions were visualized in Discovery Studio program to identify interacting amino acids. Additionally, hydrogen-bonding interactions for all ligands are noted in Additional file 1: Table S1. Two binding pockets were found to have docking scores above 40 while correlating with inhibitory activities and were considered for further analysis.

A schematic representation showing a process from GOLD Docking to predicting the final binding pocket is illustrated in Fig. 6. Binding pockets 1–22 are high binding pockets (HBP) with GOLD Scores above 40 for all ligands and binding pockets 23–28 have scores that correlate with the IC_50_ values of the ligands (ICBP).

Binding pockets 23–28 showed GOLD scores correlating with IC_50_ values; myricetin > morin > quercetin = 6-hydroxy flavonol = galangin > kaempferol. It was observed that while myricetin had the highest docking score in many cases and kaempferol the lowest, morin was close to the quercetin, 6-hydroxy flavonol, and galangin scores (Table 1).

**Table 1:** Docking scores of flavonols at potential BPs along with experimental *K*_i_ values

Ligand	*BP# 23*	*BP# 24*	*BP# 25*	*BP# 26*	*BP# 27*	*BP# 28*	Experimental *K*_i_ (μM)^a^
myricetin	44.9843	43.4749	39.7426	34.9258	33.4030	31.5181	0.85
morin	41.5695	40.0641	38.2520	31.4341	25.6152	29.3939	4.0
quercetin	40.2787	38.7066	32.9974	32.1224	26.5935	30.9113	~ 40
6-hydroxy flavonol	41.3446	41.8128	31.3729	30.6833	23.4966	31.0623	~ 40
galangin	41.7086	32.1758	31.7116	31.5844	26.8369	26.5540	~ 40
kaempferol	38.3442	26.1726	34.3741	27.1258	24.6340	24.1499	> 40

*BP* Binding Pocket

^a^Reported *K*_i_ values

## Discussion

### Analysis of ligand-interacting amino acid residues of E6 oncoprotein

**Fig. 7:**
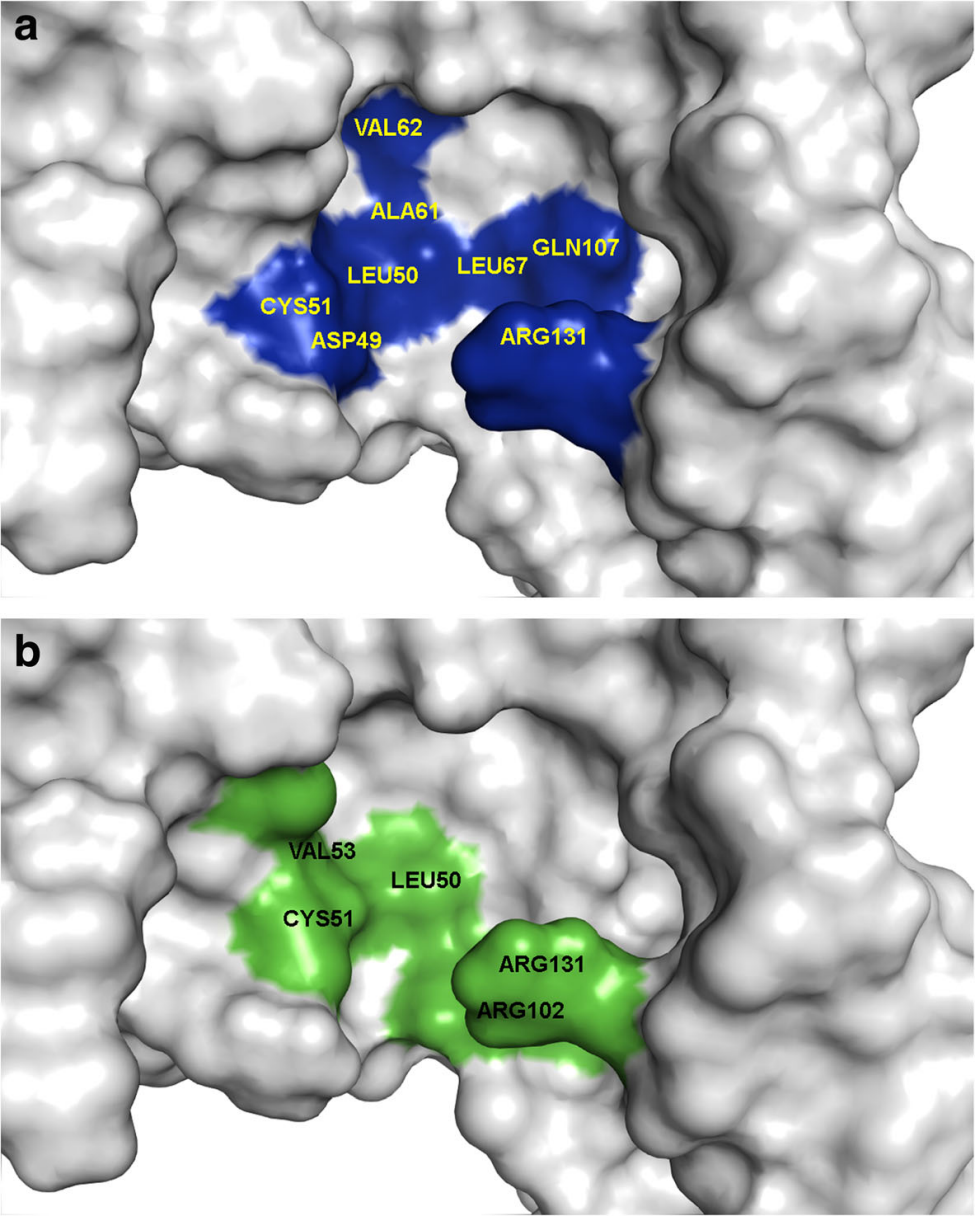
**a**) E6 oncoprotein showing amino acids common to binding pockets (blue) with docking scores correlating with IC50 values (ICBP); **b**) E6 oncoprotein showing amino acids common to binding pockets (green) with high docking scores (HBP)

After identifying the significant amino acids present in all 28 binding pockets, some trends of amino acid recurrences were observed. Cys51 was observed in 21 of 24 HBPs interacting, with at least 4 ligands followed by Leu50 seen almost as frequently. All inhibitors frequently showed hydrogen-bond interactions with Cys51, which is a critical interaction for ligand binding. Arg102 and Arg131 also are common interactions, but they were seen less frequently than Cys51 and Leu50. Val62 and Leu67 interact only with 6-hydroxy flavonol in the HBPs. Some amino acids that seemed to be specifically interacting with myricetin were Ala61 and Asp49. Amino acid residue Leu50 repeatedly showed interactions with all ligands except with myricetin in the HBPs. Among ICBPs, Cys51 and Leu50 were also commonly observed. In addition to these amino acid residues, Val53, Ala61, Arg131, and Gln107 also had a high number of interactions with the ligands in ICBPs. Unlike HBPs, Ala61 and Asp49 interacted with multiple ligands among ICBPs. Leu67 and Val62 showed interactions with all ligands except myricetin in ICBPs and also had a high number of interactions with the ligands. These amino acids may provide additional information towards their role in E6 oncoprotein inhibitory activity. Figure 7 shows common amino acids across the two sets of clusters HBPs and ICBPs. Some amino acids, however, were very specific to either of these sets and only showed in one or the other. For example, His78 and Arg129 only showed up in ICBPs. Nonetheless, these were found in clusters with low docking scores and hence were excluded from further study.

### E6 flavonol binding pocket

**Fig. 8:**
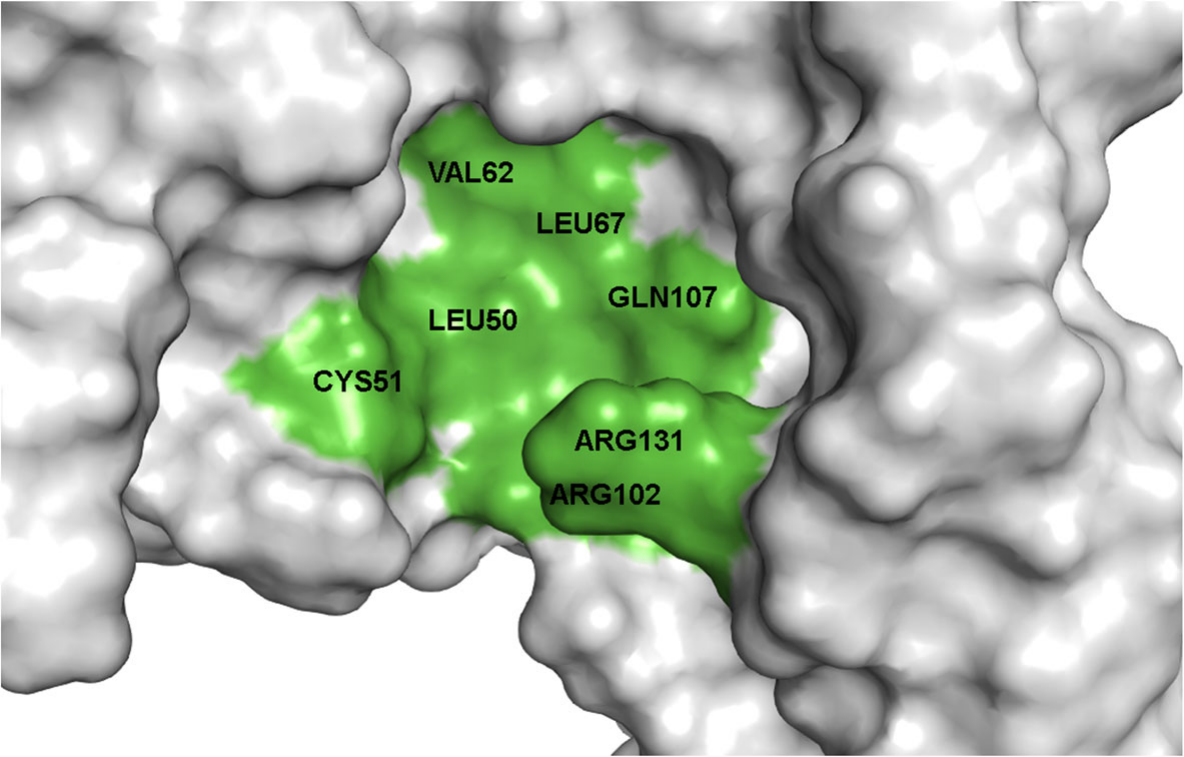
E6 oncoprotein with key amino acid residues in the potential binding pocket

**Fig. 9:**
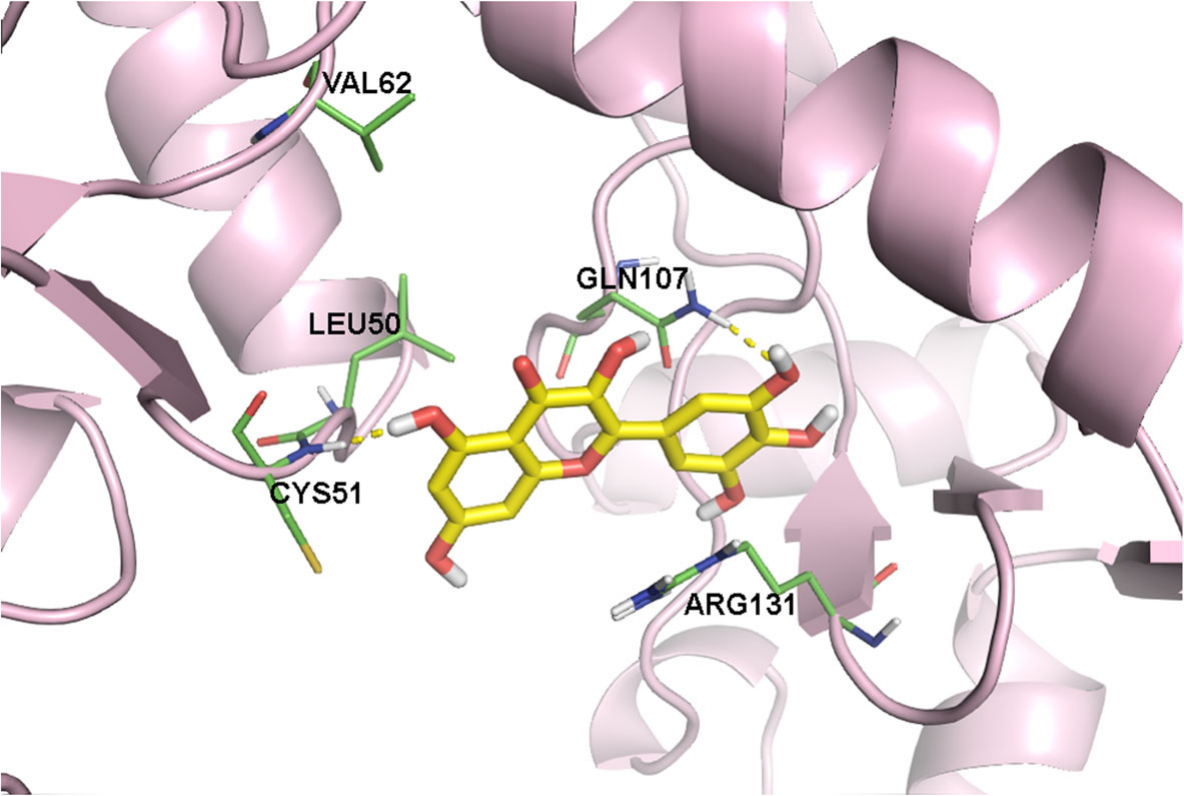
Myricetin docked into proposed binding pocket showing ligand protein interactions (also see Additional file 1: Figure S1 where myricetin showing additional H-bonding with Gln107 unlike other all other ligands at ICBP number 23. Additional file 1: Table S1 provides docking scores of ligands in all 68 identified binding pockets)

After determining receptor-ligand interactions between the E6 oncoprotein and the six known inhibitors, there are definitely amino acids that could serve as a basis for determining a potential biological active pocket for the inhibition of HPV16 E6. The amino acids Cys51, Leu50, Arg102, Arg131, Leu67, Val62, and Gln107 are seen more commonly among ICBPs, indicating their importance in E6 inhibition as shown in Fig. 8. In most cases, ligands demonstrated a hydrogen bond interaction with Cys51. Myricetin, the most potent ligand, exhibited an additional hydrogen bond with Gln107. Figure 9 shows myricetin docked into a binding pocket showing interactions with these amino acid residues.

In addition to the flavonol inhibitors of the HPV16 E6 interaction with procaspase 8 on which this study is based [20, 22], there have been other studies examining small molecule inhibitors for E6 interactions, and some other molecules have been proposed as a result of virtual screening [23–26], discovered through screening [27–30], or created by design [19, 31]. It is important to emphasize that each of these reports used assays measuring the interaction of the E6 protein with different protein partners and not with procaspase 8.

The earliest report of a small molecule inhibitor of the interaction between HPV16 E6 and the p53 tumor suppressor protein, was in 2005 for the flavone jaceosidin at a concentration of at least 10 μg/mL (~ 30 μM) [30]. This same research group reported on luteolin and wogonin, but their mechanism of action in cervical cancer cells was to suppress HPV18 E6 and HPV16 E6 expression, respectively [28, 29]. Following up on the jaceosidin lead, Kumar, et al. published two virtual screening studies using natural products or a 5000-compound library of compounds with properties similar to jaceosidin [24, 26]. Their docking study identified the E6 residues Phe54, Pro116, Leu117 and Lys122 as jaceosidin interacting residues responsible for the blockade of its interaction with p53.

The Androphy group has reported on designed inhibitors of the HPV16 E6 interaction with the ubiquitin ligase E6 associated protein (E6AP) [19, 31, 32]. Over a dozen flavone derivatives were presented and the 2, 6-substituted benzopyrazone derivative CAF-25 exhibited an IC_50_ of 2.7 mM in the HPV16 E6/E6AP binding assay and 4.4 mM in a p53 degradation assay with E6. Molecular dynamics coupled with site-directed mutagenesis defined the interactions of CAF-25 with HPV16 E6 residues Lys11, Leu50, Cys51, Leu67, Arg102, and Arg131.

The results for myricetin depicted in Fig. 9 indicate an interaction with the HPV16 E6 residues Leu50, Cys51, Gln107, and Arg131. Binding pocket amino acids that were identified using flavonols in this study do overlap to some extent with the LxxLL binding pocket amino acids [33–36]. LxxLL peptide inhibitors of E6 are known to induce apoptosis. E6 binding to LxxLL peptides interferes with the apoptotic pathway, leading to cellular proliferation. Small molecule inhibitors binding to the LxxLL binding pocket interfere with the peptide binding, subjecting the cell to apoptosis.

## Conclusions

A majority of cervical cancer patients are infected with HPV. Prophylactic HPV vaccines can only reduce or prevent cancers to some extent. These are not effective in already infected patients. Also, HPV vaccines are very expensive and may not be accessible in developing countries. Small molecule inhibitors may be the only choice in unvaccinated countries. In general, HPV16 E6 inhibitors provide a promising option for treating cervical cancers. Though the crystal structure of E6 protein is known, information about specific ligand binding pocket is unknown. In this study, the potential binding pocket that the E6 inhibitors bind was predicted. It was generally observed that Leu50 and Cys51 are necessary for high binding affinity, with Cys51 being essential for hydrogen bonding. Docking Scores of myricetin were higher than the other ligands in most of the binding pockets, which definitely agrees with experimental IC_50_ values [20].

Efficacy of the reported ligands or ligands developed for this target may also be improved through conjugation with multivalent glycocalixarenes, which are known to interact with biological macromolecules. Glycocalixarenes have demonstrated reaching viral and bacterial targets and interfere with their infections [37, 38]. E6 drugs could also be combined with other molecules targeting different cancer-specific pathways, such as molecular chaperons like HSP90 and HSP70 to treat cancer and neurodegenerative diseases [39]. For instance, the myricetin and HSP70 activators such as 115-7c were used to investigate the mechanisms underlying in Alzheimer and tau-related diseases [40].

In summary, identification of the ligand binding pocket helps to design novel inhibitors of the HPV16 E6 oncoprotein as a promising treatment for cervical cancer.

## Methods

Molecular modeling studies were carried out on a Dell Precision workstation with Intel (R) Xeon (R) CPU E5–1620 v3 @3.50GHz processor. Structure building, docking and analysis were carried out using Discovery Studio (Dassault Systèmes BIOVIA, Discovery Studio Modeling Environment, Release 2017, San Diego: Dassault Systèmes, 2016), GOLD (Genetic Optimization for Ligand Design) suite, version 5.4.0, protein ligand docking package [18–20], and the PyMOL Molecular Graphics System, Version 1.8 Schrödinger, LLC.

### HPV16 E6 crystal structure

The X-ray crystal structure of HPV16 E6 (pdb: 4GIZ) was downloaded from the Protein Data Bank and utilized for the docking experiments [33, 41]. The unit cell contains two instances of the E6 protein designated chain ‘C’ and ‘D’. For the studies described, ‘C’ was used. Preliminary ‘blind docking’, described below, either chain ‘C’ or ‘D’ provided similar results, so the differences between the two instances of the protein were considered to be insignificant.

### Blind docking

Blind docking was performed using a web-based program called SwissDock [42, 43] that predicts all potential binding sites on a target protein. SwissDock is also equipped with a database of proteins and ligand structures. It works based on a docking software named EADock DSS [43]. EADock DSS has the ability to dock in a specified region on the protein or all target cavities (blind docking). The protein structure is specified in the program using its PDB code from the Protein Data Bank [41] or by uploading structure files. The ligand files could be in the Mol2 format or CHARMM formatted files. The ligand can also be selected from the built-in ZINC database [44] or by uploading the structure. There are three docking settings that can be selected, from very fast, fast, and accurate. The amount of CPU processing time increases along with increasing accuracy. Upon submission of the docking, using the “Submit Docking” tab, it can be followed up using a URL provided. EADock DSS generates several binding modes of a ligand and the most favorable binding modes are clustered and provides an output file called “Predictions” file. Each cluster represents various conformations of a ligand at the given location on the protein and each cluster represents a pocket on the target protein. The different clusters could be viewed in the SwissDock web browser through a Jmol applet. Individual clusters were saved into separate files for further visualization using EditPlus text editing software [45].

### Visualization of protein-ligand interactions

Discovery Studio Visualizer (version 4.1.0) was used to determine protein-ligand interactions. The HPV16 E6 protein and each ligand cluster was opened in Discovery Studio. The amino acid residues that displayed interactions with the ligand were documented. Discovery Studio also shows the types of bonds with different colors and the distance between them.

### Docking

Docking into individual binding pockets was carried out using GOLD (Genetic Optimization for Ligand Docking) program. GOLD uses genetic algorithm that explores the binding pocket and searches for the best ligand interactions [46–48]. Docking studies were performed with default settings using 100 genetic algorithm (GA) runs using the GOLD score as a scoring function. Hermes visualizer in the GOLD suite was used to prepare the protein for docking. HPV16 E6 protein was prepared by adding hydrogens, removing extra water molecules and removing metals and other ligands. The receptor binding site was defined by specifying amino acid residues, which were based on the interactions noted from the blind docking in SwissDock. Default values of all other parameters were used. Higher GOLD scores represent greater affinity of the ligand for the protein at the defined binding site.

## Additional file

Additional file 1: **Figure S1.** Structural overlay of all six flavonol ligands in ICBP 23 showing the additional hydrogen bond of myricetin with Gln107. Note also that 6-hydroxy flavonol oriented in the site in the reverse direction compared to the other ligands. **Figure S2.** Sample SwissDock predictions output file for Morin docked in E6 protein. **Table S1.** GOLD scores of the six flavonol ligands in the 68 binding pockets from ‘Step 4’ in the text that indicated the presence of hydrogens bonds and other protein-ligand interactions. Sites 1-22 are high binding pockets (HBP) and 23-28 are binding pockets that correlated with the IC_50_ values for the flavonol ligands (ICBP). The best ICBP was 23 which is depicted in Fig. 9 of the text. Interacting residues are not shown for the remaining sites 29-69. The * symbol indicates that no hydrogen bond interaction was observed between the protein and the ligand. (DOCX 955 kb)

## Data Availability

We do not have any additional supporting data needed for this manuscript. We included supporting data as a part of this manuscript as a separate file.

## Abbreviations

FADD: Fas-associated death domain
Fas: First apoptosis signal;
HPV: Human papillomaviruses
pRB: Retinoblastoma protein
TNF: Tumor necrosis factor
TRADD: TNF receptor associated death domain protein
TRAIL: TNF-related apoptosis-inducing ligand
