# Supplementary material for: Identification of potential binding pocket on viral oncoprotein HPV16 E6: a promising anti-cancer target for small molecule drug discovery
Source: BMC Mol Cell Biol. 2019 Aug 6;20:30. doi: 10.1186/s12860-019-0214-3 (PMC6685234; doi:10.1186/s12860-019-0214-3)
Supplement: Supplementary file 1 — Figure S1. Structural overlay of all six flavonol ligands in ICBP 23 showing the additional hydrogen bond of myricetin with Gln107. Note also that 6-hydroxy flavonol oriented in the site in the reverse direction compared to the other ligands. Figure S2. Sample SwissDock predictions output file for Morin docked in E6 protein. Table S1. GOLD scores of the six flavonol ligands in the 68 binding pockets from ‘Step 4’ in the text that indicated the presence of hydrogens bonds and other protein-ligand interactions. Sites 1-22 are high binding pockets (HBP) and 23-28 are binding pockets that correlated with the IC50 values for the flavonol ligands (ICBP). The best ICBP was 23 which is depicted in Fig. 9 of the text. Interacting residues are not shown for the remaining sites 29-69. The * symbol indicates that no hydrogen bond interaction was observed between the protein and the ligand. (DOCX 955 kb) [file 12860_2019_214_MOESM1_ESM.docx]

**Identification of potential binding pocket on viral oncoprotein HPV16 E6: A promising anti-cancer target for small molecule drug discovery**

Srikanth Kolluru,* Rosemary Momoh, Lydia Lin, Jayapal Reddy Mallareddy and John L. Krstenansky*

**KGI School of Pharmacy, 535 Watson Dr., Claremont, CA 91711, USA**

**E-mail: Srikanth_Kolluru@kgi.edu; John_Krstenansky@kgi.edu**

**Appendix A. Supplementary Information**

**Graphical Abstract**


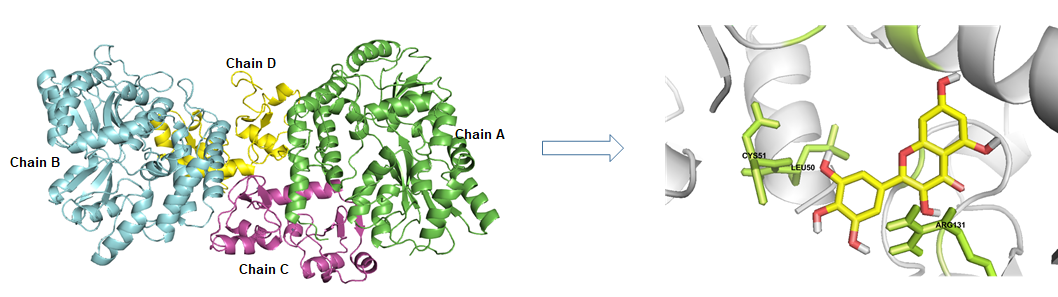


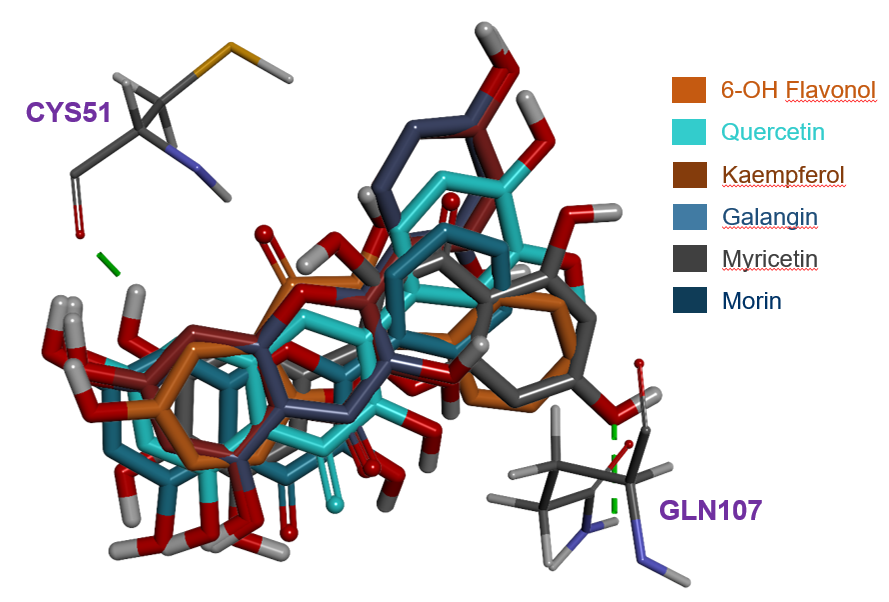


**Fig. A1** Structural overlay of all six flavonol ligands in ICBP 23 showing the additional hydrogen bond of myricetin with Gln107. Note also that 6-hydroxy flavonol oriented in the site in the reverse direction compared to the other ligands.

**
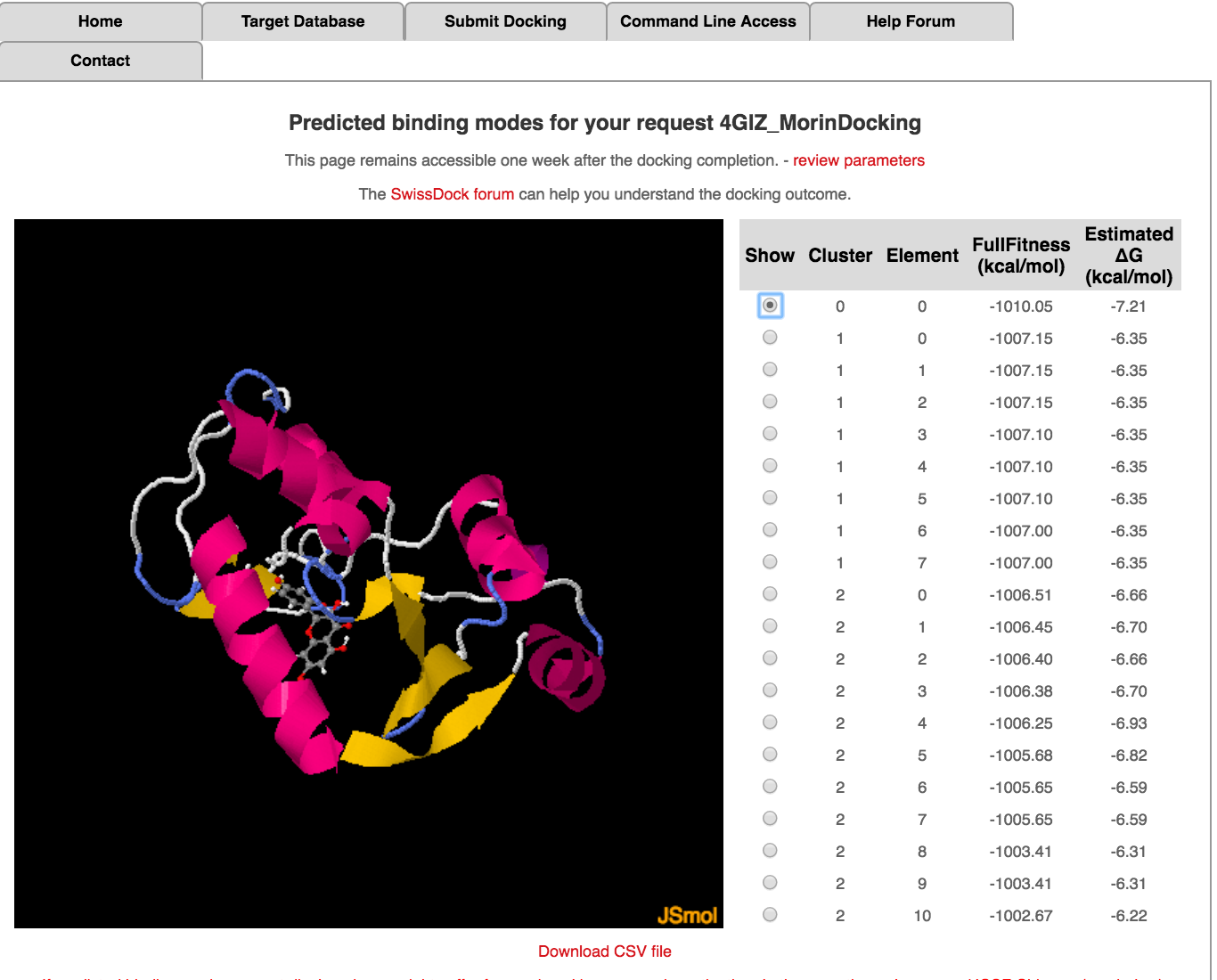
**

**Fig A2: Sample SwissDock predictions output file for Morin docked in E6 protein**

**Table 1** GOLD scores of the six flavonol ligands in the 68 binding pockets from ‘Step 4’ in the text that indicated the presence of hydrogens bonds and other protein-ligand interactions. Sites 1-22 are high binding pockets (HBP) and 23-28 are binding pockets that correlated with the IC_50_ values for the flavonol ligands (ICBP). The best ICBP was 23 which is depicted in Figure 9 of the text. Interacting residues are not shown for the remaining sites 29-69. The * symbol indicates that no hydrogen bond interaction was observed between the protein and the ligand.

| **BP #** | **E6 residues defining the binding pocket** | **Docking Score (GOLD);**  **Ligand-E6 residue interactions** | | | | | |
| --- | --- | --- | --- | --- | --- | --- | --- |
|  |  | **myricetin** | **morin** | **quercetin** | **6-hydroxy flavonol** | **galangin** | **kaempferol** |
| 1 | **CYS51 VAL62 TYR70** | 41.4951  ARG131 | 43.0771  LEU50 CYS51 ARG102 ARG131 | 45.5055  LEU50 CYS51 ARG102 | 43.0624  LEU50 | 44.0277  LEU50 CYS51 ARG102 ARG131 | 44.1761  LEU50 CYS51 ARG102 |
| 2 | **LEU50**  **CYS51 VAL62 ALA61** | 43.4036  ALA61 CYS51 ARG131 | 43.135  LEU50 CYS51 ARG131 | 45.5075  LEU50 CYS51 ARG102 | 42.1227  LEU50 VAL53 PHE45 | 44.015  LEU50 CYS51 | 43.4321  LEU50 CYS51 ARG102 ARG131 |
| 3 | **CYS51 VAL62** **LEU67** **ALA61** | 44.6671  CYS51 ASP49 ALA61 ARG131 | 43.1165  LEU50 CYS51 ARG102 ARG131 | 45.4594  LEU50 CYS51 ARG102 | 42.3249  LEU50 CYS51 VAL62 LEU67 | 44.0291  LEU50 CYS51 | 43.9918  LEU50 CYS51 ARG102 |
| 4 | **LEU50 CYS51 VAL62** | 44.5757  CYS51 ALA61 ARG131 | 42.875  LEU50 CYS51 ARG102 | 45.3282  LEU50 CYS51 ARG102 | 41.8561  LEU50 CYS51 VAL62 LEU67 | 44.0236  LEU50 CYS51 | 43.8559  CYS51 |
| 5 | **LEU50 CYS51 ARG131** **ILE128** | 44.7146  CYS51 LYS11 | 42.8859  LEU50 CYS51 | 45.0083  CYS51 ASP49 TYR60 ARG102 | 42.4609  LEU50 CYS51 VAL62 LEU67 | 43.9139  LEU50 CYS51 | 44.3866  LEU50 CYS51 ARG102 |
| 6 | **LEU50 VAL53 CYS51** **VAL62** | 43.5367  CYS51 ALA61 ASP49 ARG102 | 42.8116  LEU50 CYS51 ARG102 ARG131 | 45.478  LEU50 CYS51 | 41.9558  LEU50 CYS51 VAL62 LEU67 | 43.8001  LEU50 CYS51 | 43.0702  LEU50 CYS51 |
| 7 | **LEU50 CYS51** **VAL62** **LEU67** | 43.1791  CYS51 ALA61 ASP49 ARG102 ARG131 | 43.0672  LEU50 CYS51 ARG131 ARG102 | 45.4753  LEU50 CYS51 ARG102 | 42.2786  LEU50 CYS51 VAL62 LEU67 | 44.034  LEU50 CYS51 | 44.1119  LEU50 CYS51 ARG102 |
| 8 | **LEU50 VAL53 VAL62 TYR32** | 41.6471  CYS51 | 44.7006  LEU50 ARG102 | 44.8217  LEU50 ARG131 | 43.6206  LEU50 VAL62 VAL53 | 43.2567  LEU50 TYR60 | 44.3877  ARG102 |
| 9 | **LEU50 VAL62** **LEU67 TYR32** | 40.2836*  CYS51 ARG102 | 43.5947  N/A | 44.3039  LEU50 ARG102 | 43.4967  LEU50 VAL53 PHE45 | 43.3456  N/A | 44.5853  ARG102 |
| 10 | **LEU50 VAL53** **CYS51** | 43.4018  CYS 51 LEU50 ARG102 GLN107 TYR32 | 43.0726  CYS 51 LEU50 ARG102 ARG131 | 45.4988  CYS 51 LEU50 | 42.184  VAL53 VAL62 LEU50 | 40.7904  CYS 51 LEU50 | 42.4274  CYS 51 LEU50 |
| 11 | **LEU50 VAL53 CYS 51 TYR70** | 44.8558  ARG131 ALA61 CYS 51 | 42.95918  ARG131 LEU50 CYS 51 ARG102 | 45.44548  LEU50 CYS 51 ARG102 | 43.052  LEU50 VAL53 VAL62 | 44.03338  LEU50 CYS51 | 44.36128  LEU50 CYS 51 ARG102 |
| 12 | **LEU50 CYS51 LEU67** **TYR70** | 40.3385  ARG131 | 43.0827  LEU50 CYS51 | 45.5275  LEU50 CYS51 | 43.1195  LEU50 VAL53 VAL62 | 44.0821  LEU50 CYS51 | 44.2621  LEU50 CYS51 ARG102 |
| 13 | **LEU50 CYS51 SER71** | 44.1038  CYS51 | 42.9898  LEU50 CYS 51 ARG131 | 45.3811  LEU50 CYS51 | 43.2849  LEU50 VAL53 VAL62 | 44.0453  LEU50 CYS51 | 44.4042  LEU50 CYS51 |
| 14 | **CYS51 LEU67** | 44.844  CYS 51 ALA61 ARG131 | 43.2904  LEU50 CYS 51 ARG131 | 45.2511  LEU50 CYS 51 ARG131 | 42.8101  LEU50 | 44.0717  LEU50 CYS51 | 44.3767  LEU50 CYS51 |
| 15 | **LEU50**  **SER74** | 41.3271  ARG102 CYS51 | 43.5842*  LEU50 ARG102 | 44.0873*  LEU50 ARG102 TYR60 | 43.0479*  LEU50 | 42.862*  LEU50 | 43.2589*  LEU50 ARG102 TYR60 |
| 16 | **LEU50 CYS51 VAL62 LEU67 ALA61** | 41.8373  CYS 51 ASP49 ALA61 ARG131 | 43.7738  LEU50 CYS 51 | 44.332  LEU50 CYS 51 ARG102 ARG131 | 43.1525  LEU50 CYS 51 LEU67 VAL62 | 43.2961  LEU50 CYS 51 | 44.8347  LEU50 CYS 51 ARG102 |
| 17 | **LEU50 VAL53 VAL62 LEU67 TYR32** | 43.5148  CYS51 | 43.3092  LEU50 ARG102 | 44.7811  LEU50 CYS51 ARG102 | 44.841  VAL62 LEU50 | 42.7065 | 38.8702*  ARG102 |
| 18 | **LEU50 VAL53 CYS51 VAL62 LEU67 TYR70** | 44.605  CYS 51 ASP49 ALA61 ARG131 | 42.8232  LEU50 CYS 51 ARG102 ARG131 | 45.4178  LEU50 CYS51 | 42.1658  CYS51 VAL62 LEU50 LEU67 | 44.0503  LEU50 CYS51 | 43.847  LEU50 CYS51 |
| 19 | **LEU50 VAL53 CYS51 VAL62 LEU67** | 44.1817  CYS 51 ALA61 ARG131 | 42.8856  LEU50 CYS 51 ARG102 ARG131 | 45.4679  LEU50 CYS51 | 42.1752  CYS51 VAL62 LEU50 LEU67 | 43.8763  LEU50 CYS51 | 44.3366  LEU50 CYS 51 ARG102 |
| 20 | **LEU50 VAL53 CYS51 VAL62 TYR70** | 43.8201  CYS 51 ALA61 ARG131 | 42.8014  LEU50 CYS51 | 44.964  LEU50 CYS 51 ARG131 | 42.3874  CYS51 VAL62 LEU50 LEU67 | 43.9851  LEU50 CYS51 | 43.7875  LEU50 CYS51 |
| 21 | **LEU50 VAL53 CYS51 ARG131 LEU67** | 44.7917  LYS11 CYS51 | 43.0952  LEU50 CYS 51 ARG131 ARG102 | 45.35  LEU50 CYS 51 ARG102 | 42.9248  LEU50 VAL62 | 44.0378  LEU50 CYS51 | 44.3238  LEU50 CYS 51 ARG102 |
| 22 | **LEU50 VAL53 CYS51 VAL31 TYR32** | 45.6154  CYS 51 ALA61 ARG131 | 43.3808  LEU50 CYS 51 ARG102 | 42.6805  CYS 51 ALA61 ARG131 ARG102 | 44.2384  LEU50 VAL62 | 43.4098  LEU50 CYS51 | 44.8848  LEU50 CYS 51 ARG102 |
| 23 | **LEU50 CYS51 LEU67 ASP49** | **44.9843**  LEU50 CYS51 LEU67 ARG102 GLN107 ARG131 | **41.5695**  LEU50 CYS51 VAL62 LEU67 ALA61 GLN107 | **40.2787**  LEU50 CYS51 | **41.3446**  LEU50 CYS51 VAL62 LEU67 | **41.7086**  LEU50 CYS51 ARG102 GLN107 ARG131 | **38.3442**  LEU50 CYS51 ARG102 |
| 24 | **LEU50 CYS51 ASP49** | 43.4749  CYS51 ALA61 ARG131 | 40.0641  LEU50 CYS51 VAL62 LEU67 | 38.7066  LEU50 CYS51 VAL62 LEU67 ASP49 ARG102 | 41.8128  LEU50 CYS51 VAL62 LEU67 | 32.1758  LEU50 CYS51 ARG102 GLN107 ARG131 | 26.1726  LEU50 CYS51 ASP49 |
| 25 | **ARG131 SER74 ARG129** | 39.7426  HIS78 ARG129 ARG131 | 38.252  HIS78 ARG131 | 32.9974  HIS78 ARG129 ARG131 | 31.3729  SER74 HIS78 ARG129 ARG131 | 31.7116  HIS78 ARG129 ARG131 | 34.3741  ILE104 ILE128 ARG129 ARG131 |
| 26 | **VAL53 CYS51 VAL31** | 34.9258  CYS51 VAL31 ALA61 | 31.4341  VAL53 VAL62 VAL31 | 32.1224  LEU50 VAL53 VAL62 | 30.6833  LEU50 VAL53 VAL62 VAL31 TYR32 | 31.5844  VAL53 VAL62 VAL31 TYR32 | 27.1258  LEU50 CYS51 VAL53 |
| 27 | **SER74 ILE73** | 33.403  SER74 | 25.6152  TYR70 SER74 ARG77 HIS78 | 26.5935  SER74 ARG77 | 23.4966  TYR70 ILE73 | 26.8369 | 24.634  TYR70 ILE73 SER74 |
| 28 | **TYR70 SER71** | 31.5181  TYR32 TYR70 GLN107 | 29.3939  TYR32 TYR70 SER74 | 30.9113  TYR70 SER71 SER74 | 31.0623  LEU50 VAL62 LEU67 TYR32 TYR70 SER71 GLN107 | 26.554  TYR70 SER71 GLN107 | 24.1499  TYR32 TYR70 SER71 SER74 |
| 29 | **VAL62 TYR32** | 28.4665 | 24.1283 | 22.2441 | 21.5421 | 20.1531 | 23.6845 |
| 30 | **LEU50 CYS51 ASP49 ARG102 VAL62 LEU67** | 44.3178 | 38.7655 | 38.6214 | 40.6679 | 44.8403 | 44.1307 |
| 31 | **LEU50 VAL53 TYR32** | 39.7664 | 38.5534 | 43.8173 | 43.425 | 43.2332 | 43.0193 |
| 32 | **VAL53 CYS51 TYR70** | 39 | 42.964 | 44.9518 | 43.1283 | 44.0288 | 44.2653 |
| 33 | **ARG131 SER74 ARG129** | 39.7426* | 38.252* | 31.7116* | 31.3729* | 32.9974 | 34.3741* |
| 34 | **TYR70 CYS51** | 39.1438 | 42.9024 | 45.4371 | 42.9981 | 43.8884 | 44.2394 |
| 35 | **LEU50 CYS51 ARG102 ASP107 ARG131 LEU67** | 38.5111 | 45.9066 | 43.9453 | 42.5696 | 45.1328 | 44.6893 |
| 36 | **CYS51 VAL62** | 38.7162 | 41.6353 | 38.963 | 36.2058 | 34.5096 | 35.761 |
| 37 | **VAL53 CYS51 VAL62** | 38.9371 | 37.4714 | 36.5864 | 36.314 | 33.6668 | 41.0339 |
| 38 | **LEU50 VAL53 CYS51 VAL62 VAL31** | 37.6778 | 43.0533 | 45.2256 | 41.8141 | 43.7882 | 43.9373 |
| 39 | **LEU50 VAL53 VAL62**  **TYR32** | 37.1717 | 41.7403 | 44.816 | 43.3908 | 43.2489 | 40.5863 |
| 40 | **LEU50**  **LEU67 TYR70** | 37.2664 | 41.2216* | 44.3872* | 42.9075* | 42.8413* | 43.2036* |
| 41 | **LEU50 TYR70** | 37.4321 | 43.8262* | 44.5931* | 43.022* | 42.7049* | 43.0566* |
| 42 | **LEU50 VAL31 VAL53 VAL62 TYR32** | 37.6456 | 41.6759 | 42.5105 | 43.3225 | 44.4571 | 42.5912 |
| 43 | **LEU50**  **CYS51**  **TYR70** | 37.0515 | 42.9509 | 44.9826 | 42.9141 | 44.0405 | 44.3076 |
| 44 | **TYR32 SER74 ILE73** | 36.4495 | 34.5345 | 35.0059 | 39.5916 | 34.0555 | 34.1033 |
| 45 | **LEU50**  **VAL53 CYS51 VAL31** | 35.8692 | 42.9583 | 44.8485 | 42.2776 | 43.3581 | 43.5109 |
| 46 | **LEU50 ILE128** | 34.6182* | 44.0287* | 44.5081* | 42.949* | 42.7105* | 43.0747* |
| 47 | **CYS51**  **VAL62 LEU67** | 34.4155 | 42.8389 | 45.1781 | 43.0819 | 43.8682 | 44.1981 |
| 48 | **LEU50**  **CYS51** | 33.6362 | 40.678 | 40.9264 | 41.8128 | 26.5338 | 36.385 |
| 49 | **CYS51**  **TYR70** | 32.5248 | 42.9968 | 45.4757 | 43.018 | 44.0125 | 44.2476 |
| 50 | **LEU50**  **VAL53 VAL62 LEU67** | 30.4021 | 41.9223* | 38.8409* | 42.5505* | 42.3447* | 40.7017* |
| 51 | **SER74**  **TYR70** | 30.7113 | 29.6379 | 31.5151 | 23.6006 | 28.4266 | 30.2786 |
| 52 | **TYR32**  **ILE73** | 30.9885 | 31.7658 | 30.5787 | 29.1415 | 27.9146 | 32.0132 |
| 53 | **TYR70**  **ILE73** | 28.2593 | 31.4497 | 28.4739 | 20.3197 | 24.4512 | 24.7376 |
| 54 | **LEU50 VAL62 ALA61** | 28.3643 | 28.852 | 28.904 | -249.733* | 23.0771 | 22.2998 |
| 55 | **LEU50 VAL62 LEU67** | 28.81398* | 35.8689* | 22.5218* | 30.7933* | 34.2924* | 35.0958* |
| 56 | **LEU50 VAL53** | 26.8507 | 38.2072* | 44.08* | 43.0552* | 38.5574* | 41.314* |
| 57 | **LEU50**  **VAL62** | 22.0053 | 21.6583 | 22.0699 | -397.393 | -105.098 |  |
| 58 | **LEU50 VAL53 VAL62 VAL31** | 23.7424 | 41.1513 | 39.8385 | 43.0443 | 38.5907 | 40.3404 |
| 59 | **LEU50 CYS51 LEU67** | 23.7424 | 41.1513 | 39.8385 | 43.0443 | 38.5907 | 40.3404 |
| 60 | **LEU50 LEU67** | 21.1721 | 16.7202 | 17.2437 | 30.029 | 34.0641 | 33.277 |
| 61 | **VAL53 VAL62** | 16.8532 | 32.5427* | 32.837* | 30.5438* | 31.1082* | 31.5477* |
| 62 | **ARG131 SER71 ARG129** | 13.1884 | 38.5309* | 39.6122* | 42.5237* | 40.4812* | 40.9095* |
| 63 | **ARG131 SER71 ARG129** **SER74** | 12.7285* | 41.3468* | 44.1711* | 40.0358* | 41.3996* | 40.668* |
| 64 | **VAL53 TYR32** | 12.1181* | 38.3298* | 43.9514* | 42.5964* | 38.4245* | 40.8866* |
| 65 | **LEU50 VAL53 VAL62** | 12.3948* | 38.2625* | 39.8707* | 42.4211* | 38.7192* | 40.1194* |
| 66 | **LEU50 VAL53 VAL62 ALA61** | 11.3613 * | 41.367* | 39.4196* | 42.7225* | 38.0839* | 40.5797* |
| 67 | **VAL53**  **VAL62**  **ALA61** | 12.0682 | 32.24* | 32.288* | 30.6564* | 31.6884* | 31.3674* |
| 68 | **TYR70 ^36^ ARG131 SER71 ARG129** | 2.5577* | 41.4471* | 43.8081* | 42.7369* | 38.4415* | 38.4415* |
